# Nurses' Hours

**Published:** 1920-06-12

**Authors:** 


### Nurses' Hours.

We have received a letter on this topic from a nurse in training who signs her name, and we have been at pains to investigate the complaints she makes. We cannot, however, publish the nurse's letter, or the reply of the authorities where she is being trained, because our letter to the nurse has come back to us through the Dead Letter Office; and we cannot publish what is at present equivalent to an anonymous communication. If this lady will send us her real name and address, which will be treated in the strictest confidence, her letter shall be duly printed.—Ed., The Hospital.

